# The complete mitochondrial genome of *Chalcophora japonica chinensis* Schaufuss, 1879 (Coleoptera: Buprestidae)

**DOI:** 10.1080/23802359.2022.2113750

**Published:** 2022-08-29

**Authors:** Mingqing Weng, Yue Wang, Jing Huang, Lulu Huang, Yiqi Lin, Qilian Zheng, Yanzhen Wu, Songqing Wu

**Affiliations:** a College of Forestry, Fujian Agriculture and Forestry University, Fuzhou City, China; b Key Laboratory of Integrated Pest Management in Ecological Forests, Fujian Province University, Fujian Agriculture and Forestry University, Fuzhou City, China; c Lianjiang County Forestry Bureau, Fuzhou City, China

**Keywords:** *Chalcophora japonica chinensis*, phylogenetic analysis, complete mitochondrial genome

## Abstract

*Chalcophora japonica chinensis* Schaufuss, 1879 (Coleoptera: Buprestidae) is a common pine pest in Chongqing, Fujian, Yunnan, and other in China. The mitochondrial genome of *C. japonica* is 15,759 bp in size. The genome includes 13 protein-coding genes (PCGs), 22 transfer RNA genes (tRNAs), and two ribosomal RNA genes (rRNAs). The overall GC content of the mitogenome is 32.0%. The results showed that *C. japonica* was most related to *Chrysochroa fulgidissima*, *Trachys variolaris*, and *Agrilus mali*. The full mitochondrial genome of *C. japonica* is now available, allowing researchers to better understand the species' genetic evolution and regulatory strategies.

*Chalcophora japonica chinensis* Schaufuss, 1879 (Coleoptera, Buprestidae) is a common pine pest, usually attacking *Pinus thunbergii* Parl, *Pinus massoniana* Lamb, and other *Pinus* species. Larvae bore the tree trunk, while the adults feed on young buds and tree leaves (Song 2020). It is primarily found in Fujian, Jiangxi, Hunan, Yunnan, and other Chinese provinces (Zhang 2011) and includes two species and seven subspecies, accounting for 29% of those known in the Palearctic Region (Kubáň 2016). Wei et al. used morphological features and DNA barcodes to describe subspecies in the larval stages (Wei et al. 2020). Until now, this widely spread and hazardous species has been ignored and little research has been done. Here, we describe the whole mitochondrial genome of *C. japonica* and determined the possible phylogenetic relationships using the maximum-likelihood method and the Tamura–Nei model. These data will help researchers to better comprehend the genetic evolution of *C. japonica*.

The *C. japonica* adults were captured by trapping in Minhou county, Fujian Province, China, through the method of entrapment (118°57′15″E, 26°09′09″N). The voucher specimen (no. JD-202101) is stored in the Fujian Agriculture and Forestry University (Songqing Wu, dabinyang@126.com, URL: https://lxy.fafu.edu.cn). An adult leg was used to extract the total genomic DNA by using the TruSeq DNA Sample Preparation Kit. DNA concentration and quality were measured using a NanoDrop 2000. High-quality DNA products were isolated by magnetic bead purification. DNA was sequenced with Illumina Hiseq 2500 (Illumina, San Diego, CA). The average sequencing depth of the mitotic genome was 1937.011486. An amount of 50,889,514 clean reads was obtained after filtering from 52,428,398 raw reads; Read was made into the clean data and then MitoZ and metaSPAdes were used to be assembled (Nurk et al. 2017). Mitomaker was used to annotate the assembled sequence (Schomaker-Bastos and Prosdocimi 2018) and transfer RNA (tRNA) prediction was done with the tRNA scanning software (Lowe and Eddy 1997). GenBank accession number OM161962 was used a reference for the complete mitochondrial genome sequence of *C. japonica*. The complete mitochondria genome of *C. japonica* is 15,759 bp in size. The overall CG content of the mitogenome is 32.0%, including 38.5% A, 29.5% T, 19.6% C, and 12.4% G. The genome includes 13 protein-coding genes (PCGs), 22 tRNAs, and two ribosomal RNA genes (rRNAs). The rrnL genes size was 1281 bp and the rrnS genes size was 769 bp. Phylogenetic analysis was performed using 13 PCGs (15,759 bp), including 5253 amino acids. Except ND4 terminated by the incomplete codon T, the other eight PCGs (ATP8, ATP6, COX1, COX2, COX3, ND2, ND4L, ND5, and ND6) terminated with the codon TAA, two PCGs (CYTB and ND1) terminated with codon TAG and all PCGs began with a typical ATN codon. The phylogenetic relationships of *C. japonica* with related Coleoptera species were confirmed through the creation of an evolutionary tree. The MAFFT 7.0 program was used to compare the sequence of the *C. japonica* gene to *Anoplophora horsfieldii* as an out-group (Katoh and Standley 2013). Phylogenetic trees were generated using the maximum-likelihood method and the Tamura-Nei model of 1000 bootstrap repeats in MEGAX (Kumar et al. 2018). The data indicate that *C. japonica* is mostly related to *Chrysochroa fulgidissima*, and closely related to *Trachys variolaris* and *Agrilus mali* (Figure 1). The full mitochondrial genome of *C. japonica* is now available, allowing researchers to better understand the species genetic evolution and regulatory strategies.

**Figure 1.**
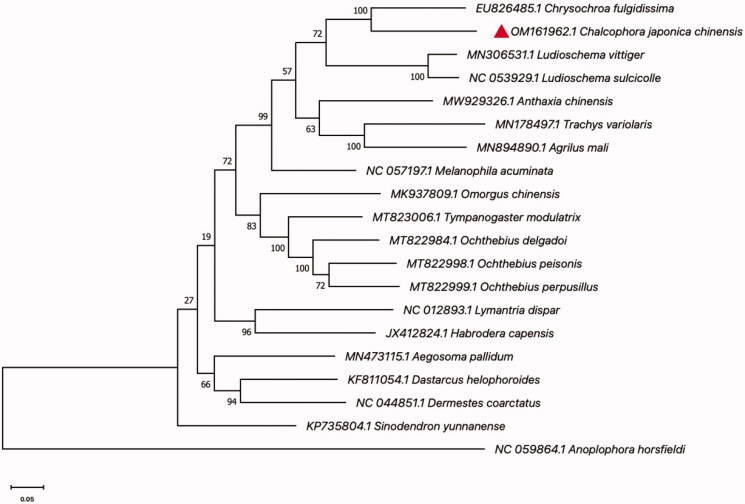
Maximum-likelihood tree of the *Chalcophora japonica chinensis* and related 19 different species of Coleoptera based on the genome sequence. Bootstrap support values are labeled near the branch.

## Ethical approval

The material involved in the article does not involve ethical conflicts. This study was permitted by the Key Laboratory of Integrated Pest Management in Ecological Forests, FAFU, China. All collection and sequencing work were strictly executed under local legislation and related laboratory regulations to protect wild resources.

## Author contributions

Conceived and designed the experiments: Songqing-Wu. Experimenters: Mingqing Weng, Yue Wang, and Jing Huang. Data analysis and interpretation: Mingqing Weng, Lulu Huang, and Yiqi Lin. Qilian Zheng and Yanzhen Wu collected the specimens. The manuscript was written by Mingqing Weng. All authors have read and agreed to the published version of the manuscript.

## Data Availability

Data supporting the findings of this study are publicly available in GenBank of NCBI at https://www.ncbi.nlm.nih.gov under the accession no. OM161962. The associated BioProject, Bio-Sample, and SRA numbers are PRJNA796309, SAMN24842196, and SRR17555532, respectively.
